# The role of photodynamic therapy on multidrug resistant breast cancer

**DOI:** 10.1186/s12935-019-0815-0

**Published:** 2019-04-11

**Authors:** Eric Chekwube Aniogo, Blassan Plackal Adimuriyil George, Heidi Abrahamse

**Affiliations:** Laser Research Centre, Faculty of Health Sciences, University of Johannesburg, P.O. Box 17011, Doornfontein, Johannesburg, 2028 South Africa

**Keywords:** Breast cancer, Multidrug resistance, *P*-glycoprotein, Photosensitizer, Photodynamic therapy

## Abstract

Breast cancer heterogeneity allows cells with different phenotypes to co-exist, contributing to treatment failure and development of drug resistance. In addition, abnormal signal transduction and dysfunctional DNA repair genes are common features with breast cancer resistance. Chemo-resistance of breast cancer associated with multidrug resistance events utilizes ATP-binding cassette (ABC) efflux transporters to decrease drug intracellular concentration. Photodynamic therapy (PDT) is the treatment that involves a combination of a photosensitizer (PS), light and molecular oxygen to induce cell death. This treatment modality has been considered as a possible approach in combatting multidrug resistance phenomenon although its therapeutic potential towards chemo-resistance is still unclear. Attempts to minimize the impact of efflux transporters on drug resistance suggested concurrent use of chemotherapy agents, nanotechnology, endolysosomal release of drug by photochemical internalization and the use of structurally related compound inhibitors to block the transport function of the multidrug resistant transporters. In this review, we briefly summarize the role of membrane ABC efflux transporters in therapeutic outcomes and highlight research findings related to PDT and its applications on breast cancer with multidrug resistance phenotype. With the development of an ideal PS for photodynamic cancer treatment, it is possible that light activation may be used not only to sensitize the tumour but also to enable release of PS into the cytosol and as such bypass efflux membrane proteins and inhibit escape pathways that may lead to resistance.

## Introduction

Breast cancer is the most frequent cancer amongst women and a serious public health problem all over the world. It is a dominant cause of female morbidity and mortality [1]. Global statistics as of 2017 from the American Cancer Society (ACS), estimated 252,710 and 2470 new cases of breast cancer will be diagnosed among women and men respectively. The ACS estimates that approximately 40,610 women and 460 men are expected to die from breast cancer in the same year. Breast cancer incidence and death rates generally increase with age but vary greatly in survival rates due to availability of early detection and treatment methods among racial/ethnic groups [2]. Current treatments for breast cancer include; surgery, chemotherapy, immunotherapy and radiation therapy [3]. The eradication and therapeutic success of breast cancer are related to tumour stratification and dissemination patterns classified into four stages based on size, age, node involvement and tumour grade. These stages are 1; consists of well-defined and localized tumour mass, characterized by poor invasion properties. Stage 2 and 3, corresponds to an increased tumour volume and acquisition of invasive phenotype. The metastasis dissemination and a huge tumour size with invasive phenotype are classified as stage 4 [4]. Chemotherapy, radiation and targeted therapies have made major advances in patient management over the past decades but refractory diseases and recurrence remain common [5]. This is partly due to drug resistant chemotherapy caused by over expression of efflux transporters that pumps out and decreased intracellular drug accumulation [6]. Similarly, compensatory signalling also influence the molecular mode of resistance where cancer cells uses alternative pathways to escape treatment and inhibits cell death [7]. Taking this in consideration, breast cancer biology and its regulation, impact of efflux transporters and the role of photodynamic therapy on cancer therapeutic outcomes as well as multidrug resistance mechanism are discussed below.

## Lifestyle risk factors and implications in breast cancer

Breast cancer research in the past 25 years has established many risk factors that involve genetic and behavioural factors. However, risk increases with germline and somatic mutation in the BRCA 1 and BRCA 2 genes, among other exposure to irritant carcinogenic agent that disrupts the immune and hormonal signalling, thus leads to inflammation and cancer [1]. Further research into the changes in form and appearance of epithelial cells in the mammary gland of women with cancer have revealed more evidence about the environmental lifestyle changes that initiate tumour progression. Lifestyle changes include: excessive alcohol intake, tobacco smoking as well as exposure to chemical agents or ionizing radiation. All these factors contribute to an increase in frequency of mutations and induce uncontrolled cell proliferation and metastasis through molecular interaction with proteins involved in transcriptional regulatory mechanisms [1, 8].

## Breast cancer biology and transcriptional regulation

Breasts are made up of connective, glandular and fatty tissues that have lobes, lobules, ducts, areola and a nipple. These organ consist of a uniform structure of epithelial cells that secrete and produce milk after childbirth. Whenever there is a morphologic or functional alterations within its uniform epithelial structures, tumour initiation develops and later form a mass of multiple population of cells capable of evading physiological cell death [9].

The changes in gene expression patterns seen in breast cancer have provided evidence of epigenetic, genetic or post-translational altered expression of certain proteins, like transcription factors, co-regulators, and histone enzymes that order DNA into structural units according to recent study [10–12]. These proteins play a crucial role in the expression of genes that results in susceptibility of a healthy cell transformation malignant cell [10–12]. Among the first altered transcriptional regulation found in breast cancer were the overexpression and gene amplification of oestrogen receptor alpha (ERα) and avian myelocytomatosis viral oncogene homologue factor (c-myc). These two oncoproteins were found to be associated with abnormal cell division and replication within the breast [13, 14]. Different array activity reports of transcription factors in breast cancer have also shown the involvement of Twist, Snail and Slug master factors in the final epithelial–mesenchymal transition and metastatic phenotypic characteristics [15].

Additional studies have identified inherited/acquired altered gene expression as a detectable cause of carcinogenesis of breast tissue [16]. This arise after a study of some essential genes involved in cellular processes and maintenance were found to be mutated at germ cell level [17]. Next generation sequencing analysis also found higher penetrance mutations in breast cancer 1 (BRCA1), tumour protein p53, mitogen-activated protein kinase 1 (MAP3K1), retinoblastoma 1 (RB1), phosphatidylinositol-4,5-bisphosphate 3-kinase catalytic subunit alpha (PIK3CA) and GATA binding protein 3 (GATA-3) genes that results in breast cancer formation [18, 19].

## Breast cancer biomarkers and drug resistance

Several breast cancer biomarkers have been identified of which the estrogen receptor (ER), progesterone receptor (PR) and human epidermal growth factor receptor 2 (HER2) constitute the main markers. These markers represent therapeutic targets and may also play important roles in diagnosis and prediction of prognosis [20]. Their expression closely correlates with differences in tumor behavior and therapeutic responses for example, positive expression of either ER or PR is termed hormone receptor positive (HR+) breast cancer. This tumor type will likely respond and receive endocrine therapy, while HER2+ breast cancers will receive HER2 targeted therapies. A negative expression of these biomarkers is called triple-negative breast cancer (TNBC) which comprises 15–20% of all breast cancers [21, 22]. TNBC is the most serious type of tumor and its molecular classification is characterized by a negative profile of ER, PR, and HER2 [23]. According to Shaheen et al. [22], these receptors helps in targeted therapy and effective treatment of breast tumors. Histopathologic features of this tumor include a high nuclear mitotic activity with a high nuclear-cytoplasmic ratio that accelerate its proliferation and make its’ metastases highly difficult to recognize, hence referred to as metastases with unknown origin [23]. Research have found multi-drug resistance associated genes and their products, abnormal cell signal transduction, DNA repair abnormality genes as well as cell cycle checkpoint kinase 2 (CHEK2) gene dysfunction as factors closely associated with TNBC drug resistance [24]. Pro-inflammatory cytokines derived from either dying cells or tumor micro-environment may play a role in the development of TNBC resistance to therapy. Xu et al. [25] reported that TGF-β contributes to TNBC development of drug resistance through apoptotic, stemness and epithelial-to-mesenchymal transition regulation [25]. The standard of care for TNBC is neoadjuvant chemotherapy which can increase the chances of developing drug resistance. Studies by Kim [26] and his colleagues conducted at single-cell genomic DNA and RNA sequencing level revealed that TNBC harbored many residual tumor cells with clonal differences. Their data suggests that TNBC have pre-existing resistant clonal cells or adaptively selected resistant cells acquired by reprogramming in response to chemotherapy [26].

## The multidrug cancer resistance

**Fig. 1:**
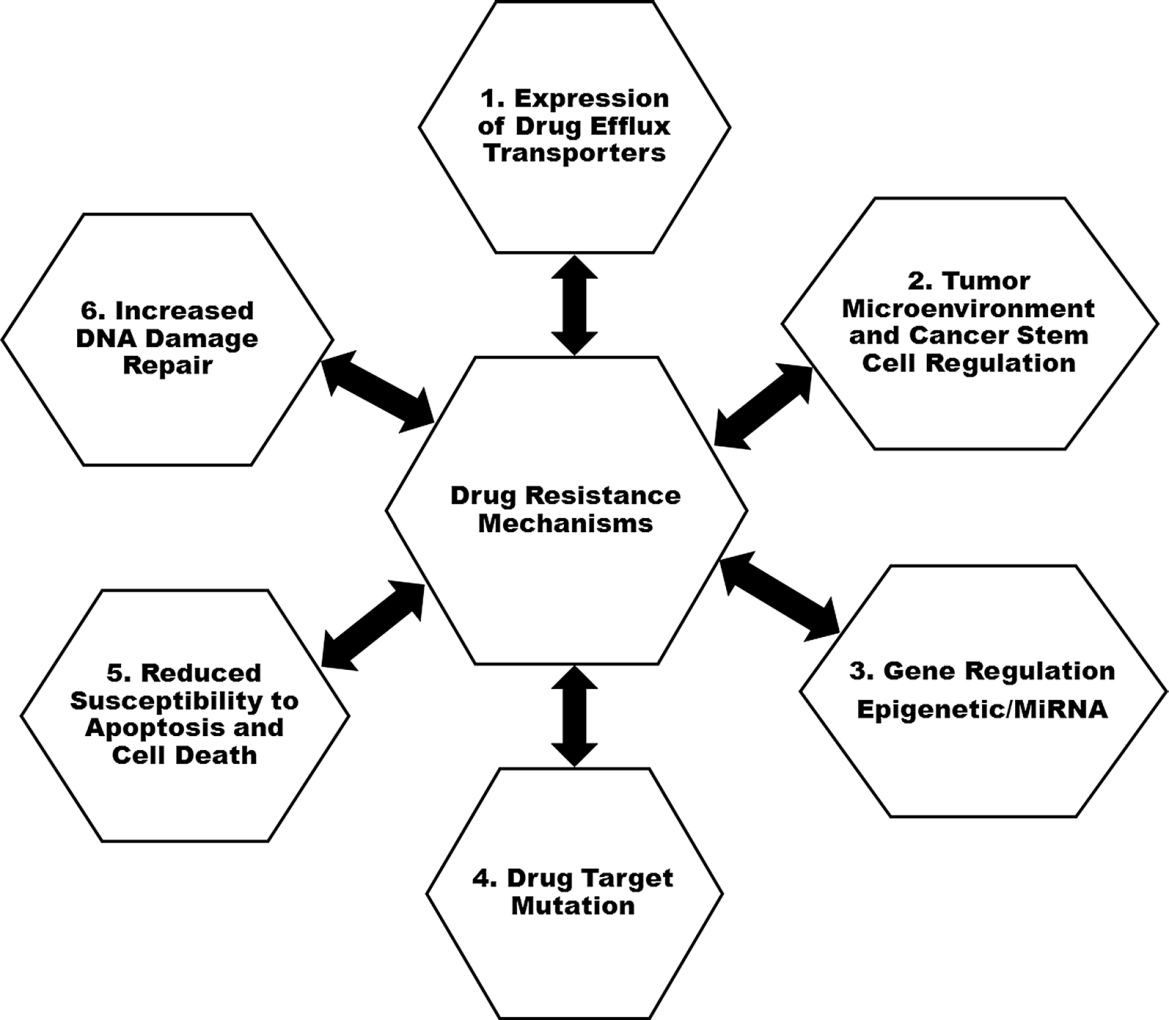
Hallmark of multidrug cancer resistance mechanisms. Tumour cells developed resistance through; increased expression of drug efflux transporters, microenvironment tumour regulation, increased epigenetic microRNA regulations, drug target modification, altered apoptotic signalling pathway and increased DNA repair mechanism

Resistance often follows initial response to chemotherapy and has remained a problem to cancer therapy. Multidrug resistance (MDR) is a phenomenon that involve a multitude of factors highlighted in Fig. 1, where certain tumour cells has the ability to evade the cytotoxic effects of a broad range of structurally and functionally unrelated drug.

This phenomenon can be intrinsic; when cancer cells show innate ability to resist drug treatment at initial exposure or, acquired; when cancer cells gain resistance through the active efflux of drugs during chemotherapy [9, 27].

Various mechanisms attributed to MDR includes; (1) Increased expression of drug efflux transporters; where the proteins of ATP-binding cassette family acts to protect cells by ejecting a wide variety of anticancer drug, rendering the cell resistant. (2) Changes in tumour microenvironment and cancer stem cell regulation; tumour microenvironment that comprises of stromal cells, extracellular matrix, cytokines and growth factors, all contribute to direct cell interaction mediated by drug resistance. (3) Elevating adaptability by epigenetic and microRNA regulation; hyper-methylation of oncogenes and demethylation of drug resistance genes leads to acquisition of resistance [28]. (4) Altered drug target; associated with rapid down-regulation or mutation of drug targets lead to drug structure modification in the protein, improper binding and eventually drug resistance. (5) Reduced susceptibility to apoptosis and cell death; avoidance of apoptosis through increased expression level of B cell lymphoma (BCL) family proteins that block apoptotic signalling pathways is among the important resistance mechanism of cancer cells. (6) Increased DNA damage repair system; damaged DNA or replication errors are continuously detected and fixed by DNA proofreading and repair mechanisms (Fig. 1) [29]. Further classifications are based on cellular mechanisms which are grouped into classical transporter and non-transporter-based MDR phenotypes. Different ABC transporters identified in a human genome have been classified into seven subgroups (A–G) based on sequence similarities and structural organization [30]. This classification grouped four members of classes (A, B, C and G) as classic transporters capable of conferring drug resistance [31]. The classic transporters including *p*-glycoprotein (*P*-gp, gene symbol ABCB1), breast cancer resistance proteins (BCRP, gene symbol ABCG2) and multidrug resistance-associated proteins (MRP, gene symbol ABCC1) enables the cells to efflux anticancer drug thus, decreasing the intracellular concentration of the drug [32, 33]. The process called efflux-transport mechanism is mainly associated with the overexpression of the ATP-binding cassette proteins which protects the cell in physiological conditions, by forming a unique defence network against cellular toxicants [9, 34]. It is generally believed that ABC transporters possess multiple drug binding sites in a large pocket formed by transmembrane α-helices that facilitates transportation across membrane in a competitive-dependent manner [35].

## Multidrug resistance mediated by efflux transporters

**Fig. 2:**
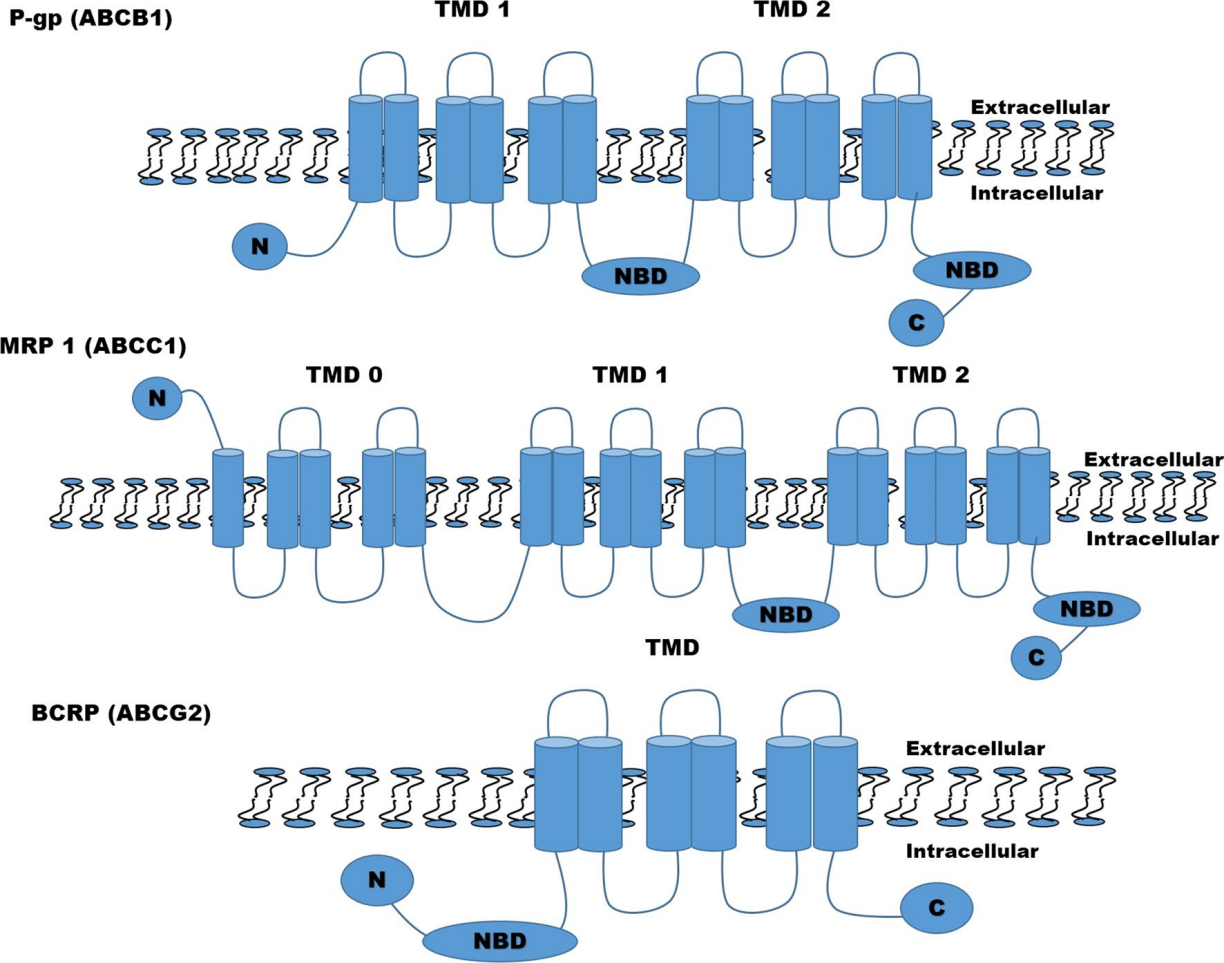
ATP-binding cassette transporters *P*-glycoprotein (*P*-gp), multidrug resistance-associated protein 1 (MRP1), breast cancer resistance protein (BCRP). *P*-gp consists of 2 nucleotide binding domains (NBD) and 2 transmembrane domains (TMDs). MRP1 structure has 2 TMDs and 2 NBDs. It also has a third TMD (TMD0) with 5 transmembrane segments and an extra N-terminus. While BCRP is formed by only 1 NBD and 1 TMD. The TMD consist of 6 transmembrane fragments which is asymmetrically located in 2 membranes and facilitates transcellular transport of a drug or metabolites from intracellular to extracellular or vice versa within the cell

Multidrug resistance mediated by drug efflux ATP-binding cassette (ABC) transporters emerged in the 1970s as an important phenomenon to explain/account for the clinical resistance of cells to standard chemotherapy. With the identification of other drug efflux pumps, it’s now clear that chemo sensitivity and resistance are governed by these transporters as well as other genetic and environmental factors [9, 36, 37]. Importantly, rapid up regulation of efflux transporters was reported to contribute in decreased intracellular anticancer drug accumulation thereby precluding the therapeutic efficacy and consequently MDR. The main features and structure of *P*-gp, MRP and BCRP with respect to cancer and their cellular localization are illustrated in Fig. 2.

## *P*-glycoproteins (*P*-gp/ABCB1)

One of the three well-characterized transporters associated with chemo-resistant mechanisms of a variety of drugs is *P*-glycoprotein. It’s a transmembrane glycoprotein molecule with the size of 170-kDa that acts as an energy-dependent efflux transporter [38]. It consists of 1280 amino acids constituted by two transmembrane domains (TMDs), each consisting of six transmembrane segments and two nucleotide binding domains linked with N- and C-termini (Fig. 2) [9, 39, 40]. *P*-glycoprotein, an energy-dependent export transporter was the first human ABC transporter identified in 1976 [41]. This protein is known to transport and efflux a different variety of hydrophobic compounds including cancer drugs [31, 42]. *P*-gp is prominently expressed in the epithelial cells of mouse and human tissues at the physiological barriers such as the blood–brain barrier, gastrointestinal tract, kidney and liver [43]. Its location at the apical membrane of endothelial cells enable its protective effect. The overexpression of this protein, associated with MDR has led to the identification of many important drugs that can serve as substrate that bind and enhance its transport function [44]. *P*-gp have a high flexible drug binding sites that enable its interaction with hundreds of structurally diverse chemical compounds, including anticancer drugs, steroid hormones and hydrophobic toxic peptides [45]. Another important feature of *P*-gp is that it recognizes and transports hydrophobic drugs or substrates thus suggesting lipid membrane partitioning as an essential step for its transport [39]. Despite understanding of the structure and cellular localization of *P*-gp, its precise molecular mechanism of drug transport is still not fully understood. Nevertheless, several hypothetical models like hydrophobic vacuum cleaner and lipid flippase activity have tried to explain the mechanism of substrate efflux by *P*-gp. According to the vacuum cleaner model of *P*-gp function, the drug/substrate are partitioned into the membrane and are spontaneously translocated into the cytoplasmic leaflet where it gains access to the *P*-gp substrate binding sites from within the bilayer interior and subsequently effluxes into the extracellular environment. In the lipid flippase activity model, drugs/substrates are flipped to the outer membrane leaflet after gaining access to the *P*-gp substrate binding sites. Both activity models cause dimerization of the two nucleotide binding domains and thus ATP hydrolysis which returns the protein back to its inward facing drug binding conformation and reinitiates the transport cycle [39, 46]. In tumour cells that express *P*-gp, this would result in reduced intracellular concentrations, which decreases the cytotoxicity of several anticancer agents. However, there are possibilities that there might be other complementary mechanisms that are directly related to anticancer drug efflux which can confer resistance. Studies have demonstrated correlation between elevated *P*-gp expression and patient response rate following chemotherapy. Trock and colleagues [47] examined *P*-gp expression in patients with breast cancer after administration of chemotherapy and the study showed a threefold likelihood of patients with over expression of *P*-gp not to respond to chemotherapy than other patients. Other studies by Triller et al. [48] and Leith et al. [49] contradicted the report of Trock and his team by demonstrating that there is no relationship between patient’s response to therapy and *P*-gp expression.

## Multidrug resistance-associated protein (MRP)

Phylogenetic analysis of human genome have identified and characterized 45 human ABC genes, which have been grouped into seven subfamilies: A–G. The subfamily C group comprises of nine proteins often referred to as MRPs (multidrug resistance proteins) or ABCC proteins [50]. This transporter protein utilizes the power of ATP binding and hydrolysis to move their physiological and pharmacological substrates across the plasma membrane [51]. There are two types of MRPs; the short (MRP4, MRP5, MRP8 and ABCC12) and long (MRP1, MRP2, MRP3, ABCC6 and MRP7). Both types contain the ABC structure with two nucleotide and three transmembrane domains (Fig. 2). The TMDs contain the transmembrane helices that form the translocation pathway through which substrates are transported across the membrane. These transport proteins have different substantial transport kinetics with a degree of overlapping substrate specificity. MRP’s transport a large number of molecules derived from exogenous and endogenous sources across the plasma membrane except for the proteins encoded by the ABCC6 and ABCC12 genes that are not known to transport drugs; hence they are not referred to as MRP6 and MRP12. Each MRPs have their unique pharmacological and physiological functions as well as differences in tissue distribution and membrane localization. Some in vitro studies have established that MRP1 is responsible for resistance to many widely used anticancer drugs; hence it is considered the most clinical relevant MRPs [52]. This 190-kDa transmembrane protein is mainly found and distributed in all organs conferring resistance of cell to several structurally unrelated cytotoxic agents across blood-organ interfaces [53]. Later after MRP1 was discovered to confer multidrug resistance, Leier and co-workers [52] also discovered that MRP1 have a high affinity for pro-inflammatory cytokine leukotriene (LTC4). The study by Wijnholds et al. [54], confirmed the key physiologic role of MRP1 in mediating LTC4 export. Their study together with Ishikawa [55] demonstrated that MRP1 was (at least one of) the ubiquitous ATP-dependent GSH conjugate pumps that facilitates the transport of substance across membrane. Additionally, reports from Slot et al. [51] have also demonstrated the role of MRP2 and MRP4 in the elimination process of xenobiotics and their metabolites from the bile and urinary system. Equally, if not important, these xenobiotic-transporting proteins have contributed to the pharmacokinetics profiles, efficacy and toxicity of a large number of therapeutic and diagnostic agents found in the environment and diet [51].

## Breast cancer resistance protein (BCRP)

Breast cancer resistance protein is a polytopic transmembrane (TM) protein with 655 amino acids. It’s the second member of the subfamily G of the large human ATP-binding cassette (ABC) superfamily with gene symbol ABCG2 according to HUGO nomenclature [35]. Whereas in CD (clusters of differentiation) nomenclature, it was assigned the term CD338 by the Human Cell Differentiation Molecules Organization [36]. Unique distinguishing features of BCRP from other efflux transporters like *p*-gp and MRP1 are on its structure which only have one nucleotide-binding domain that precedes one membrane spanning domain (Fig. 2). These domain organizations are opposite to that of *P*-gp and MRP1 which accounts for differences in its transport mechanism [56]. The BCRP can function as homo or heterodimer with molecular mass ranging from 72 to 180 kDa [57]. Biological research of ABC family proteins revealed that BCRP is normally expressed in the gut, bile canaliculi, blood brain barriers, placenta and the renal proximal tubular cells where they function as a defence mechanism to protect tissue against xenobiotic exposure as well as contribute to the absorption, distribution, and elimination of drugs and endogenous compounds [35]. The role of BCRP in drug disposition has become an area of interest because of its high resemblance with *P*-gp in tissue, and also because of its distribution and expression with similar substrate and inhibitor specificity (Table 1) [35]. Over the past two decades, BCRP protein have been under intense study to demonstrated its role in drug resistance to anthracycline anticancer drugs on MCF-7 cell line in the absence of overexpression of known multidrug resistance transporters such as *P*-gp or MRP1 [58]. Doyle and his team reported a direct involvement of BCRP in natural resistance and longevity of normal stem cells. Their report leads to the assessment of any relationship between BCRP expression and clinical outcomes in breast cancer and other solid tumours. Burger and his co-workers [59] found a positive correlation between BCRP mRNA expression and response to patients receiving anthracycline-based chemotherapy in breast cancer. Alternative study also examined BCRP expression and its resistance to 5-fluorouracil (a BCRP substrate) in 140 breast cancer tissue specimens, and found that resistance to 5-fluorouracil was significantly correlated with the levels of BCRP expression [60]. Further correlation between high levels of BCRP expression and poor clinical outcomes particularly in acute myeloid leukaemia have also been reported [36]. Even though the role of BCRP in drug resistance in cancer has not been well established, its role as an active efflux transporter on drug absorption, distribution, metabolism and excretion has been understood [36]. A variety of PS including pheophorbide A, protoporphyrin IX, and related compounds have been identified as BCRP substrates [61]. However, several studies now are looking towards overcoming cancer drug resistance using BCRP inhibitors.

**Table 1:** Selected substrates and inhibitors of *P*-gp/ABCB1, MRP/ABCC1, and BCRP/ABCG2 as chemosensitizers

Efflux protein transporters	Substrate	Inhibitors	References
*P*-gp/ABCB1	5-Fluorouracil, doxorubicin, paclitaxel, vincristine, vinblastine, vindesine, vinorelbine, mitoxantrone, topotecan, actinomycin D	Cyclosporin A, quinine, verapamil, valspodar, tariquidar, zosuquidar, laniquidar, dexverapamil, nifedipine, quinidine, chlorpromazine	[39, 45, 93, 96, 97]
MRP/ABCC1	Daunorubicin, imatinib, doxorubicin, melphalan, chlorambucil, saquinivir, vincristine, irinotecan, ciprofloxacin, mitoxantrone	Biricodar/VX-710, cyclosporine A, efavirenz elacridar/GG918/GF120918, verapamil, agosterol A, curcumin, disulfiram, flavonoids, clotrimazole, steroid analogues, probenecid	[53, 96, 98–100]
BCRP/ABCG2	Mitoxantrone, camptothecin derivatives, methotrexate, lamivudine, prazosin, cimetidine, nilotinib, nitrofurantoin, flavopiridol, gefitinib	Cyclosporine A, sirolimus, tamoxifen, omeprazole, piperine, novobiocin, dofequidar, nelfinavir, boceprevir, fluconazole, dipyridamole	[35, 96]

## Breast cancer stem cell in therapeutic resistance and relapse

Accumulating evidence now suggest that human cancers including breast, lungs, cervical, leukaemia, among others are driven by a subset of cells with the capability of self-renewal ability to generate and differentiate into a functional mature progeny. These cells are known as cancer stem cells and were first isolated from acute myeloid leukaemia by John Dick and colleagues [62]. The cellular hierarchy and organization within the breast are structured in a way that stem cells generate all progeny and terminally differentiated cells with specialized functions of milk production. In breast cancer, a subpopulation of cells that displayed stem cell properties was identified and characterized by cell surface markers CD44 expression and are thus called “breast cancer stem cells” (BCSCs) [62]. Subsequent to identification of BCSCs in primary mouse xenografts model, a small population of the cells have shown to be more invasive than the differentiated cells which comprise the tumour bulk. More evidence now suggest that these cells contribute to cancer relapse following treatments [63]. The relative resistance associated with BCSCs appears to be multifactorial ranging from decreased level of oxidants production and increased DNA repair efficiency that help maintain their stemness. Moreover, the preferential targeting of rapidly dividing cells by most chemotherapy enables the BCSCs in their quiescent non-cycling state to persist after therapy [64, 65]. Another molecular mechanism mediating breast cancer resistance to trastuzumab chemotherapy is inactivation of the tumour suppressor PTEN, which activate the downstream Akt molecule and bypass HER2 activation [62].

## Unique mechanism of photodynamic therapy

**Fig. 3:**
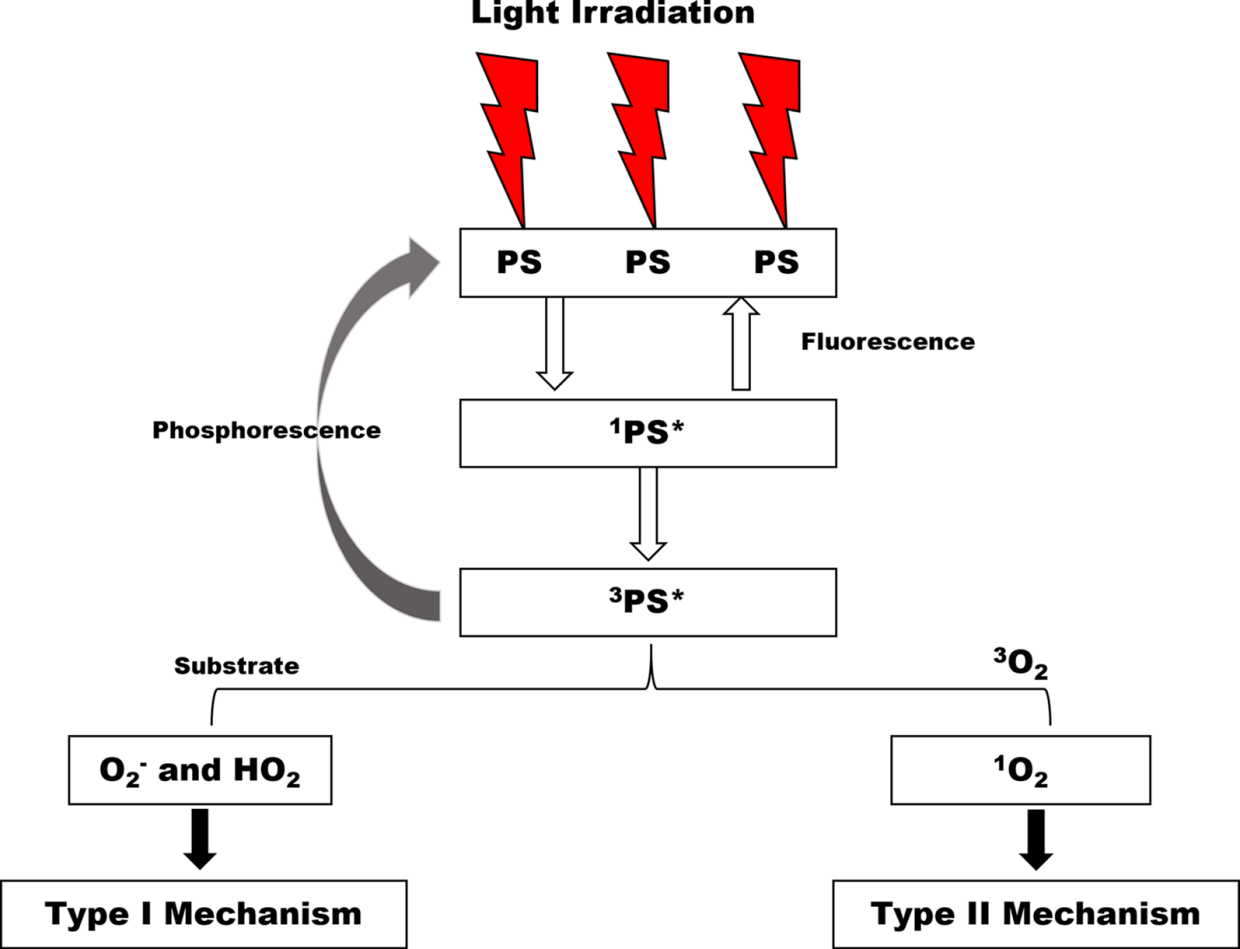
The photosensitization process of PDT. When PS absorbs photon energy, it transits from ground singlet state (PS) to an excited singlet state (^1^PS*) which then undergoes internal conversion and changes to a triplet state (^3^PS*). The triplet PS reacts with either tissue substrate (Type I mechanism) to form a superoxide anion radicals or with molecular oxygen to form a reactive oxygen species (Type II mechanism)

Photodynamic therapy is an approved treatment regime for several cancer types that involves systemic use of a non-toxic light sensitive compound (PS) and a subsequent light excitation of the PS by an appropriate wavelength to induce cancer death [66]. This treatment modality requires three components; PS, light and molecular oxygen to exert a cytotoxic effect. The PS absorbs energy from light in the form of photon and undergo energy transfer to either tissue substrate (Type I reaction) or molecular oxygen (Type II reaction) which results in the production of superoxide anion radicals and reactive singlet oxygen molecules respectively (Fig. 3) [67, 68].

The consequence of excess ROS production will cause vital tissue peroxidation and initiation of cell death mechanisms [69, 70]. The photo-damaging effect of PDT greatly depends on factors like type of PS used, dose administered, light exposure, light fluency, oxygen availability, sensitizer localization, drug administration time interval and many more [71]. It has been observed that following PDT, there are blood vessel occlusion, collapse and ultimate vascular shutdown due to excessive radical formation and hypoxia. This causes apoptosis and necrosis [72]. Moreover, PDT can also mediate destruction of tumour-infected cells through immune modulation. The radical formation results in cell signal transduction that activate apoptotic proteins and cytokine gene expression [73].

Although PDT is a site-directed therapy, its efficacy that depends on excess ROS production that can directly kill tumour cells and/or cause inflammatory immune response with tumour vasculature shutdown [74]. A major challenge in PDT technology is to acquire the therapeutic relevant PS level and retention in the target tissue. This is because of the presence of overexpressed multidrug resistance proteins among tumour cells which pumps out PS and prevents its localization. This upregulation of multidrug resistance protein especially *P*-gp have been described as the most important resistance mechanisms. The cytoprotective functions of some intracellular antioxidants like the glutathione system, catalase, lipoamide de-hydrogenase, and superoxide dismutase which detoxify PDT-induced ROS, result in treatment resistance [69]. A major cause of cancer development is the escape of T-cell recognition thus, PDT have shown to induce T-cell mediated anti-tumour immunity [75]. Reports have shown PDT to be specific treatment modalities with fewer side effects. More several research effort and strengths have been focused to demonstrate the advantages of PDT in overcoming MDR.

## The role of photodynamic therapy in overcoming multidrug cancer resistance

**Fig. 4:**
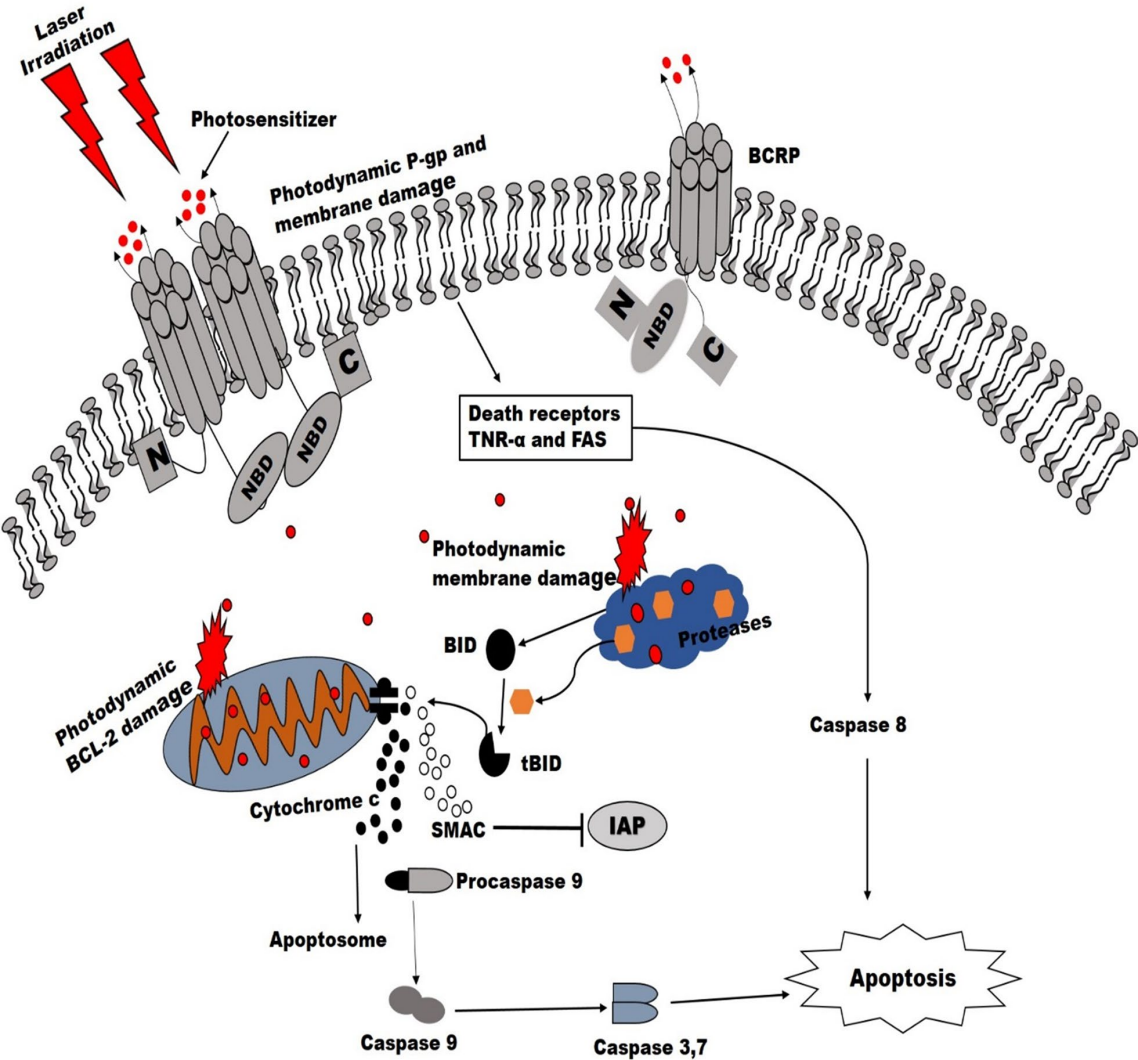
Overview of unique mechanisms of PDT-induced apoptosis on multidrug resistant cells. Light activation directly damages drug efflux pumps (*P*-gp and BCRP) involved in classical drug resistance and release PS into the cytosol which localizes on mitochondria and lysosome. Upon activation, damages the antiapoptotic BCL-2 family proteins and lysosomal membrane. The Lyso-PDT induces the proteolytic activity that cleaves BID to tBID and leads to mitochondria pore opening via BAX action. The pore opening caused the release of cytochrome c and SMAC (second mitochondrion-derived activator of caspases) from the intermembrane mitochondrion space. The SMAC promotes caspase activation by binding with IAPs (inhibitor of apoptosis protein) and cytochrome c forms complex which leads to cell death through caspase action. Membrane damage after PDT leads to depolarization, reduction of active transport and lipid peroxidation which help in activation of death signal and thus cell death

Emerging evidence now suggests that the damage and unique mechanism of photodynamic treatment on tumour and its microenvironment could possibly inhibit drug resistance pathways and re-sensitize resistant cells to standard therapies. Photoactive compound used in PDT localize at cellular organelle such as the mitochondria, lysosome, endoplasmic reticulum and possibly Golgi apparatus within the cytoplasm [76]. Upon photo-damage, these intracellular membranes including their protein components are destroyed thus leading to cell death via any of the normal modes—necrosis, autophagy or apoptosis. The photo-damage of PDT via lysosome (Lyso-PDT) and mitochondria (mito-PDT) are the most common and well-studied. PS that localized in the lysosome leads to spillage of proteases upon irradiation which activates the proapoptotic factor BID (tBID) that enhance cell death. Whereas mito-PDT damage antiapoptotic proteins of the BCL-2 family. This causes BAX translocation to the outer mitochondria membrane and stimulates the release of cytochrome c that drives the cells alone the irreversible path to apoptosis (Fig. 4) [7].

This mechanism of apoptotic induction bypasses many regulatory checkpoints that accounts for resistance and triggers increased susceptibility of tumour cells to death rather than MDR development. Some investigations have shown that photo-destruction of breast cancer resistant protein rich extracellular vesicles could facilitate photoactive drugs towards reaching its target without been entrapped or sequestrated outside the cell [34]. This approach results in direct damage to proteins involved in drug resistance and shut down tumour microvasculature, thus stimulates drug delivery and antitumor immunity [34, 77]. The time interval between PS administration and photo-irradiation which is very unique to PDT can be utilized and exploited depending on the pharmacokinetics of the PS to shut down tumour microvasculature [7].

Photochemical internalization (PCI) is another novel technological approach used to facilitate the cytosolic delivery of macromolecular drugs. This drug and gene therapy delivery method is developed to release macromolecules into the cytosol and by so doing, bypass the efflux pumps proteins that transport xenobiotics out of the cancer cells. The PCI treatment is based on same principles of PDT except in its aim which is to induce cancer cell death by the macromolecular drug delivered and not primarily by photochemical reaction [78]. PCI has been demonstrated to facilitate the intracellular release of anticancer agents or PS that are targeted for intracellular organelles. Emerging evidence support the therapeutic potential of PCI to circumvent mechanisms associated with resistance towards chemotherapeutics [78]. Furthermore, none of the PCI components including a macromolecular drug, amphiphilic PS and light are subjected to cellular efflux which enables PCI a treatment strategy for cancer stem-like cells [79].

Additionally, several researchers have reported in vitro experimental evidence of PDT potentiation in overcoming MDR and re-sensitizing the susceptibility of tumour cells to treatment. One of such studies includes early investigations by Kusuzaki and colleagues [80] that studied the effect of PDT using acridine orange on mouse osteosarcoma cells with MDR phenotype and observed a strong cytotoxic effect [80]. Similarly, Kulbacka et al. [81] used the FDA approved Photofrin PS on MDR adenocarcinoma cells and observed a comparable oxidative alterations in both sensitive and resistant cells. Also, MDR Jurkat/A4 leukaemia cells showed reduced sensitivity and no cross-resistance to ALA-mediated PDT [82]. Another study using same ALA PS with different cell line, MCF-7 MDR phenotype also showed a less effective treatment in comparison with MCF-7 parental cells [83]. Feuerstein and co-workers tested the effect of a novel ALA-derived prodrug on MCF-7 resistant sublines and the result showed a higher potent effect on the viability of the resistant cells even without laser irradiation. This indicate that ALA-derived prodrug based PDT has the effectiveness of treating resistant cancer malignancies [84]. Another most recent report by Chen et al. [85] indicated that PDT mediated by meso-5-[*p*-diethylene triaminepenta acetic acid-aminophenyl]-10,15,20-triphenyl-porphyrin (DTP) have a significant effect on Adriamycin-resistant breast cancer cells to an extent of recovering its sensitivity to Adriamycin. Chen and colleagues [85] also suggested that DTP-PDT could exhibit inhibition of MDR1 gene expression at molecular level with an important realistic significance. More also, Kukcinaviciute et al. [86] demonstrated the usefulness of mTHPC-mediated PDT on 5-fluorouracil resistant human colorectal cancer cells. These studies highlights the role of PDT in chemo-resistance reversal and potentiation in MDR.

In recent years, application of Nano-carriers and targeting technology to overcome MDR has been recognized as an important and promising field of research. Current research are now beginning to focus on the use of nanotechnology to deliver PS to specific target cells/tissues in an attempt to mitigate problems associated with poor selectivity and tumour targeting of the PS. This involve the use of drug delivery system loaded with PS to bypass the efflux transporters and enhance intracellular accumulation [87]. The use of Nano-carriers, such as polymeric nanoparticles, and magnetic nanoparticles can facilitate delivery of PS without been entrapped by efflux transporters [88]. A novel nanoceria-mediated drug delivery nanocomposites, synthesized by Hong and colleagues was used to load PS for targeted PDT. They reported that the nanocomposites carrying the PS selectively accumulated in lysosome triggered production of reactive oxygen species and reduced *P*-gp expression. This approach promotes the effectiveness of PDT in the treatment of drug-resistant human breast cancer cells [88]. Drug delivery system combined with targeting technology holds great potential and may provide the possibilities of targeting at gene level, the proteins responsible for MDR [45, 78, 89].

## Modulation of multidrug resistance

Novel strategies to modulate MDR in cancer cells including targeting ABC transporters using substrates and inhibitors are currently underway to eliminate and suppress drug resistance [45]. At molecular level, microRNA and RNA interference including synthetic siRNAs are extensively been used to reverse multidrug resistance by inhibiting the expression of genes associated with MDR. For instance, Bao and colleagues were able to modulate multidrug resistance in human breast cancer using miR-298. Their study observed that overexpression of miR-298 down-regulated *P*-gp expression, and increases nuclear accumulation of doxorubicin and cytotoxicity in resistant breast cancer cells [38].

Another more achievable approach is the use of anti-MDR strategies which include MDR inhibitors or substrates [45]. MDR transporters especially *P*-gp have proven to interact with various structurally unrelated compounds classified as substrates and modulators. This substrate actively binds to and is transported in and out of the cell while modulators bind and block the transport function of the MDR transporter. The anti-MDR strategy of using an inhibitor to alter the function of the transporter proteins have shown significant clinical applications in cancer chemotherapy [90]. There are three different generations of inhibitors developed for MDR transporter up till date; first generation inhibitors including verapamil and cyclosporine A were found to reverse drug resistance profile in leukemic and lungs cancer cells respectively [91, 92].

Its low therapeutic response and unacceptable toxicity drive the development of the second generation of inhibitors such as dexverapamil, valspodar and biricodar citrate which showed a better tolerability but displayed unwanted pharmacokinetic interaction with cytochrome P450 [45, 93]. Continuous problems with MDR necessitates the development of a specific and more potent three generation MDR transporter inhibitor that can reverse MDR with almost no pharmacokinetic interaction with other chemotherapeutic drugs. This inhibitors include; tariquidar (XR9576), zosuquidar (LY335979), laniquidar (R101933) and elacridar (F12091) [45, 93, 94]. Recent studies have noted that tariquidar can also act as a substrate depending on its in vivo dosage to *P*-gp [95]. Another achievable approach to circumvent drug resistance besides the use of ABC transporter inhibition, is by substituting drugs that are not subject to efflux transport system. Kathawala and his colleagues [44] in their report postulated that ABC transporter inhibitor known as chemosensitizers may be used in combination with standard chemotherapy to enhance therapeutic efficacy.

## Conclusion and future perspectives

Developing therapeutic strategies for breast cancer, especially the type characterized by lack of ER, PR, and HER2 expression, have been a challenge since these receptors are involved in targeted therapy. TNBC exhibit a higher risk for drug resistance and cancer relapse. In recent years, the therapeutic effects of PDT in cancer treatment are encouraging especially in palliative end point. This remains an area of interest with the possibility of serving as an alternative to broad spectrum antibiotic-based therapy and thus limits the development of drug resistance. The treatment modality of PDT is still questioned by some scientists mostly its efficacy in huge and metastases widespread tumour. The targeted delivery of PS to diseased cells is still a challenge in PDT. Since PS that accumulates in malignant tissue are crucial to photo-medicine, it is essential that more research should focus on the development of a suitable PS composed of either antibody or nanoparticles to enhance efficiency and reduce the chances of drug efflux pumps. This will further compensate the lack of specificity and selectivity potential of a raw PS. Recent advancements have shown combination treatment strategies comprising PDT and other concurrent treatments to have a better response in cancer recurrence. The improved response was due to molecular response, boosted antitumor immunity and susceptibility of cancer cells following PDT which leads to improve overall treatment outcome. Immunotherapy using drug delivery system could also be used to bypass the efflux transporters and deliver PS into tumour cells thus maximize treatment efficacy and thwart survival mechanism in resistant tumour. In addition to this development, PDT resistant cells should also be used as a model to further study the impact of PDT on the cellular targets. Moreover, PDT studies on MDR tumour cells with focus on the multidrug resistant phenotype on PS uptake might shed light and contribute to the circumvention of drug resistance. It is expected that this review will hopefully stimulate innovative preclinical and clinical PDT research against multidrug resistance cancer cells.

## Abbreviations

ABC: ATP-binding cassette
ABCB1: *P*-glycoprotein gene symbol
ABCC1: multidrug resistance protein gene symbol
ABCG2: breast cancer resistance protein gene symbol
ACS: American Cancer Society
AKT: protein kinase B
ALA: aminolevulinic acid
ATP: adenosine triphosphate
BAX: Bcl-2 associated X protein
BCL: B cell lymphoma
BCRP: breast cancer resistance protein
BCSCs: breast cancer stem cells
BID: BH3 interacting domain death agonist
BRCA1: breast cancer 1
BRCA2: breast cancer 2
CD: cluster of differentiation
CHEK2: cell cycle checkpoint kinase 2
C-Myc: avian myelocytomatosis
DNA: deoxyribonucleic acid
DTP: meso-5-[*p*-diethylene triaminepenta acetic acid-aminophenyl]-10,15,20-triphenyl-porphyria
ER: oestrogen receptor
ERα: oestrogen receptor alpha
FDA: Food and Drug Administration
GATA-3: GATA binding protein 3
GSH: glutathione
HER2: human epidermal growth factor receptor 2
HR+: hormone receptor positive
HUGO: Human Genome Organisation
IAPs: inhibitor of apoptosis protein
kDa: kilodalton
LTC4: leukotriene cytokine
MAP3K1: mitogen-associated protein kinase 1
MCF-7: breast cancer cell line
MDR: multidrug resistance
MRP: multidrug resistance-associated protein
mTHPC: meta-tetra(hydroxyphenyl)chlorin
NBDs: nucleotide binding domains
PCI: photochemical internalization
PDT: photodynamic therapy
*P*-gp: *P*-glycoprotein
PIK3CA: phosphatidylinositol-4,5-bisphosphate 3-kinase catalytic subunit alpha
PR: progesterone receptor
PS: photosensitizer
PTEN: phosphatase and tensin homolog
P53: tumour suppressor protein
RB1: retinoblastoma 1
RNA: ribonucleic acid
ROS: reactive oxygen species
SMAC: second mitochondrion-derived activator of caspases
TGF-β: transforming growth factor beta
TM: transmembrane
TMDs: transmembrane domains
TNBC: triple negative breast cancer
